# Role of Aberrantly Activated Lysophosphatidic Acid Receptor 1 Signaling Mediated Inflammation in Renal Aging

**DOI:** 10.3390/cells10102580

**Published:** 2021-09-28

**Authors:** Yongjie Jin, Eun Nim Kim, Ji Hee Lim, Hyung Duk Kim, Tae Hyun Ban, Chul Woo Yang, Cheol Whee Park, Bum Soon Choi

**Affiliations:** 1 College of Medicine, The Catholic University of Korea, Seoul 06591, Korea; kimyg1118@sina.com; 2 Transplant Research Center, The Catholic University of Korea, Seoul 06591, Korea; kun0512@hanmail.net (E.N.K.); didsuai@hanmail.net (J.H.L.); scamph@catholic.ac.kr (H.D.K.); deux0123@catholic.ac.kr (T.H.B.); yangch@catholic.ac.kr (C.W.Y.); cheolwhee@hanmeil.net (C.W.P.); 3 Division of Nephrology, Department of Internal Medicine, College of Medicine, The Catholic University of Korea, Seoul St. Mary’s Hospital, Seoul 06591, Korea; 4 The Institute for Aging and Metabolic Diseases, College of Medicine, The Catholic University of Korea, Seoul 06591, Korea; 5 Division of Nephrology, Department of Internal Medicine, College of Medicine, The Catholic University of Korea, Eunpyeong, St. Mary’s Hospital, Seoul 03312, Korea

**Keywords:** lysophosphatidic acid receptor 1, nuclear factor-κB, inflammation, aging, kidney

## Abstract

The increasing load of senescent cells is a source of aging, and chronic inflammation plays a pivotal role in cellular senescence. In addition, senescent renal tubular epithelial cells are closely associated with renal aging. Lysophosphatidic acid (LPA) is a bioactive lipid mainly produced by the catalytic action of autotaxin (ATX), and its ligation to LPA receptor-1 (LPAR1) is associated with chronic inflammation and renal fibrosis; however, its role in renal aging is unclear. Male 2-, 12-, and 24-month-old C57BL/6 mice and Human renal proximal tubular epithelial cells (HRPTEpiC) were used in the present study. DNA damage and oxidative stress-induced senescence were simulated using doxorubicin (DOXO) and H_2_O_2_, respectively. The aged kidney showed decreased renal function, increased fractional mesangial area, and tubulointerstitial fibrosis. Both aged kidney and senescent cells showed increased levels of LPAR1, Nuclear factor κB (NF-κB), and inflammatory cytokines. In addition, *LPAR1-*knockdown reduced NF-κB and subsequent inflammatory cytokine induction, and *NF-κB*-knockdown resulted in decreased LPAR1 expression. Our study revealed a positive feedback loop between LPAR1 and NF-κB, which reinforces the role of inflammatory response, suggesting that blocking of aberrantly activated LPAR1 may reduce excessive inflammation, thereby providing a new possible therapeutic strategy to attenuate renal aging.

## 1. Introduction

The progressive loss of physiological integrity is a characteristic feature of aging, which leads to impaired function and increased mortality [1]. The aged kidney shows structural and functional changes, which render it more vulnerable to injuries and pose challenges in the subsequent recovery, leading to an increased risk of developing acute kidney injury and subsequent chronic kidney disease [2,3,4]. In addition, older age is associated with poor renal replacement therapy outcomes compared to younger age [5,6,7]. In China, the prevalence of chronic kidney disease (CKD) in men and women is parallel with age, increasing from 5.1% and 7.4% in those aged 18–39 years, to 18.5% and 24.2% in those aged 70 years or older, respectively [8]. Although the prevalence of CKD varies among different countries and populations, the trend is consistent with age; thus, it poses a considerable global health burden [9,10,11].

The accumulation of senescent cells with age is considered an important promoter of the aging process [12]. Freund et al. [13] reported that senescent cells are a source of chronic inflammation. Prolonged and persistent inflammation in the aging process is damaging and destructive, and this phenomenon is referred to as “inflammaging” [14,15]. During aging, secreted inflammatory cytokines reinforce the senescent phenotype through several positive feedback loops [16]. In addition, they produce an inflammatory cascade that affects the surrounding cells. The altered intracellular and intercellular communication results in an aberrant accumulation of senescent cells, which causes or exacerbates aging and age-related diseases [13,15,17]. NF-κB nuclear binding activity has been reported to consistently increase in aged kidneys [18]. Accumulating data strongly suggest that NF-κB signaling is a major inducer of inflammatory cytokines and is continually required to maintain senescence [16,19,20].

Recently, lysophosphatidic acid (LPA)-mediated inflammation has started gaining importance. Benesch et al. [21] reported that sustained LPA-mediated signaling is an emerging hallmark of chronic inflammation, which may lead to the propagation of the disease phenotype. LPA is a bioactive lipid produced mainly by the catalytic action of autotaxin (ATX). Extracellular LPA acts through cognate six G-protein-coupled receptors (GPCRs) called LPA receptors (LPAR) 1–6, and mediates various cellular effects, such as cell survival, proliferation, metabolism, and inflammation [22,23]. A series of studies on human renal transplant biopsies and aging mouse models suggest that tubular epithelial cells (TECs) appear to be primarily responsible for renal aging [24,25,26]. LPAR1, LPAR2, and LPAR3 are the primary LPARs in normal mouse kidneys, and LPAR1 and LPAR3 are mainly located in TECs [27]. Moreover, LPAR1 is frequently implicated in renal tubulointerstitial fibrosis, and the latter is a typical feature of aged kidneys [2,28]. Kanehira et al. [29] reported that LPAR1 may promote cell senescence, whereas LPAR3 may exert the opposite effect. It has also been reported that increased LPAR1 may activate NF-κB [30,31]. All the evidence suggests that LPAR1 signaling seems to be involved in renal aging, but there is no research on this hypothesis to date.

## 2. Materials and Methods

### 2.1. In Vivo Experiments

The Animal Care Committee of Catholic University approved the experimental protocol. Aging male C57BL/6 mice were purchased from the Korea Research Institute of Bioscience and Biotechnology (Chungcheongbuk-do, Republic of Korea). Mice were housed at a controlled temperature in a controlled light environment. Mice were divided into three groups as follows: 2-month-old group (2 M group, n = 7), 12-month-old group (12 M group, n = 7), and 24-month-old group (24 M group, n = 7).

### 2.2. Cell Culture and In Vitro Experiments

Human renal proximal tubular epithelial cells (HRPTEpiC) (ScienCell, Carlsbad, CA, USA) were grown in an epithelial cell medium (ScienCell, Carlsbad, CA, USA) containing epithelial cell growth supplement (ScienCell, Carlsbad, CA, USA), in a humidified atmosphere of 95% air and 5% CO_2_ at 37 °C. HRPTEpiC was used at passages 8–10. Cells were plated at a density of 3 × 10^5^ cells/well in 6-well plates and incubated for 3 days. Fresh medium containing doxorubicin (DOXO) (Sigma, St. Louis, MO, USA) was added to cells and cultured for 24-h to induce cellular senescence. Similarly, HRPTEpiC was treated with fresh medium containing H_2_O_2_ (Sigma, St. Louis, MO, USA). Cells were harvested at the end of the treatment for further analysis.

### 2.3. Renal Function

Mice were placed in individual mouse metabolic cages (Tecniplast, Gazzada, Italy), with access to water and food for 24-h, to collect urine samples for subsequent analyses of albumin and creatinine concentrations. The presence of albuminuria (Albuwell M, Exocell, Philadelphia, PA, USA), urine creatinine levels (Abcam, Cambridge, UK), and serum creatinine concentrations (Abcam, Cambridge, UK) were determined using ELISA kits. Creatinine clearance was calculated using the following standard formula:

$$\frac{\mathrm{urine}\;\mathrm{creatinine}\;(\mathrm{mg}/\mathrm{dL})\;\times \;\mathrm{urine}\;\mathrm{volume}\;(\mathrm{mL}/24\;h)}{\mathrm{serum}\;\mathrm{creatinine}\;(\mathrm{mg}/\mathrm{dL})\;\times \;1440\;(\min/24\;h)}$$

### 2.4. Histological and Microscopic Analyses

Kidney samples were fixed in 10% formalin. The tissues were embedded in low-temperature melting paraffin, and 4 μm-thick sections were processed and stained with periodic acid-Schiff (PAS) and Masson’s trichrome. The glomerular volume and mesangial area were determined by examining the PAS-stained sections, and the relative mesangial area was expressed as the fractional mesangial/glomerular surface area. Tubulointerstitial fibrosis was defined as a matrix-rich expansion of the interstitium with tubular dilatation, tubular atrophy, tubular cast formation, and thickening of the tubular basement membrane. Ten fields were assessed per section. Data after analysis of all sections were assessed using ImageJ software (Wayne Rasband, National Institutes of Health, MD, USA).

### 2.5. Immunohistochemistry

Deparaffinized tissue sections were processed for immunohistochemistry (IHC), as described elsewhere [32], using primary antibodies against LPAR1 (Santa Cruz Biotechnology, Dallas, TX, USA). Data after the analysis of all sections were assessed using ImageJ software.

### 2.6. Immunofluorescence Analysis

Immunofluorescence analysis was used to analyze the expression of LPAR1 (Santa Cruz Biotechnology, Dallas, TX, USA) and NF-κB (Santa Cruz Biotechnology, Dallas, TX, USA) in HRPTEpiC. Cells were plated into a six-well plate at a density of 3 × 10^5^ cells/well and DOXO (100 nM) and H_2_O_2_ (100 μM) for 24-h in a humidified atmosphere of 95% air and 5% CO_2_ at 37 °C. The cells were then incubated with the primary antibody at 4 °C overnight, followed by Alexa 488 and Alexa 594 conjugated anti-mouse secondary antibodies (Invitrogen, Carlsbad, CA, USA) according to the manufacturer’s protocol. DAPI (Invitrogen, Carlsbad, CA, USA) was used for nuclear counterstaining.

### 2.7. Western Blot Analysis

Total protein was extracted from the kidney tissues using Pro-Prep Protein Extraction Solution (iNtRON Biotechnology, Gyeonggi-Do, Republic of Korea) according to the manufacturer’s instructions. For NF-κB expression, nuclear proteins were prepared using the NE-PER nuclear and cytoplasmic extraction kit (Thermo Fisher Scientific, Rockford, IL, USA). Western blot analysis was performed using the following antibodies: ATX (Proteintech Group Inc., Rosemont, IL, USA), LPAR1 (Santa Cruz Biotechnology, Dallas, TX, USA), LPAR3 (Gene Tex, Inc., Irvine, CA, USA), PI3K (Abcam, Cambridge, UK), Akt (Cell Signaling Technology Inc., Danvers, MA, USA), phospho-Akt (Sre473) (Cell Signaling Technology Inc., Danvers, MA, USA), NF-κB (Santa Cruz Biotechnology, Dallas, TX, USA), TGF-β (R&D Systems, Minneapolis, MN, USA), TNF-α (Proteintech Group Inc., Rosemont, IL, USA), IL-1β (Cell Signaling Technology Inc., Danvers, MA, USA), IL-6 (Proteintech Group Inc., Rosemont, IL, USA), GAPDH (Santa Cruz Biotechnology, Dallas, TX, USA), and Lamin-B1 (Cell Signaling Technology Inc., Danvers, MA, USA).

### 2.8. Senescence-Associated β-Galactosidase (SA-β-Gal) Staining

To detect senescent cells, we performed SA-β-gal staining using a senescence β-galactosidase staining kit (Cell Signaling Technology Inc., Danvers, MA, USA). HRPTEpiC was rinsed with PBS and fixed with a fixative solution provided with the SA-β-gal kit for 15 min at room temperature (25 °C). The plates were washed twice with PBS, β-galactosidase staining solution was added, and HRPTEpiC was incubated with the staining solution at 37 °C without CO_2_ in a dry incubator for 24 h. SA-β-gal-positive cells were detected using light microscopy.

### 2.9. Small Interfering RNA (siRNA) Transfection in HRPTEpiC

Scrambled siRNAs targeting *LPAR1* and *NF-κB* were purchased from Bioneer (Daejeon, Republic of Korea). We cultured HRPTEpiC in six-well plates for transfection, and when cells reached 60% confluence, we transfected the cells with siRNA duplex-Lipofectamine^TM^ RNAiMAX complexes (Invitrogen, Carlsbad, CA, USA) according to the manufacturer’s instructions.

### 2.10. Statistical Analysis

Data are expressed as mean ± standard error (SE). Differences between groups were examined for statistical significance using a *t*-test (SPSS). Statistical significance was set at *p* < 0.05.

## 3. Results

### 3.1. Renal Function and Histological Changes

**Figure 1:**
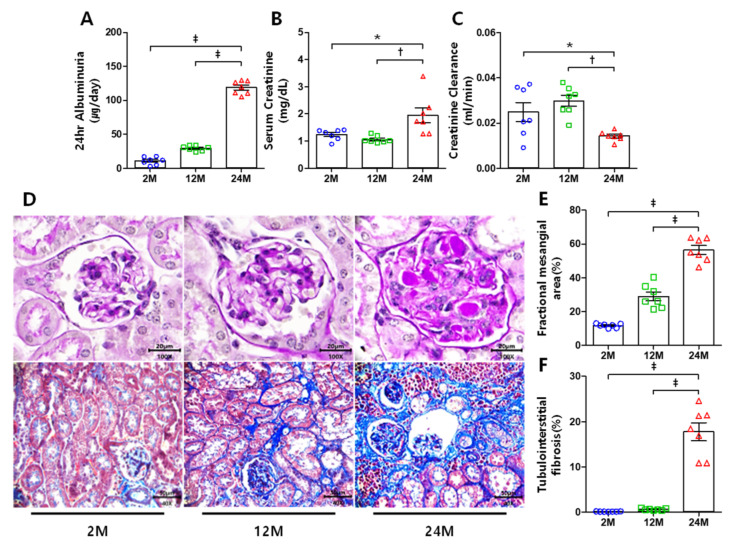
Renal functions and histological changes: (**A**) albuminuria significantly increased in the aged group. (**B**) Serum creatinine levels increased in the 24 M group compared to the 2 M and 12 M groups. (**C**) 24 M group creatinine clearance decreased compared with 2 M and 12 M groups. (**D**) Representative images of renal tissue stained with PAS (original magnification, ×1000) and Masson’s trichrome (original magnification, ×400). (**E**) Fractional mesangial area was significantly expanded in the 24 M group. (**F**) Highly increased tubulointerstitial area in the 24 M group. * *p* < 0.05, † *p* < 0.01, ‡ *p* < 0.001.

To evaluate renal function, we determined albuminuria, serum creatinine levels, and creatinine clearance in each group. Compared to the 2 M and 12 M groups, the 24 M group showed a marked increase in 24-h albuminuria excretion (Figure 1A). Similarly, the serum creatinine level increased in the 24 M group compared to that in the 2 M and 12 M groups (Figure 1B). However, creatinine clearance decreased in the 24 M group compared to that in the other groups (Figure 1C). Histological examination showed that the fractional mesangial area was significantly expanded in the 24 M group compared with the 2 M and 12 M groups (Figure 1D,E). Additionally, the 24 M group showed a dramatically increased tubulointerstitial fibrosis area compared to the other groups (Figure 1D,F). It is important to recognize that limited by tubular creatinine secretion [33] and detection method creatinine clearance may not be reliable when estimating glomerular filtration rate [34]. However, the differences among each group are consistent with histological changes, so creatinine clearance still indirectly reflects changes in renal function.

### 3.2. ATX and LPAR1 Expression Increases in Aged Mice Kidney, but LPAR3 Expression Decreases

**Figure 2:**
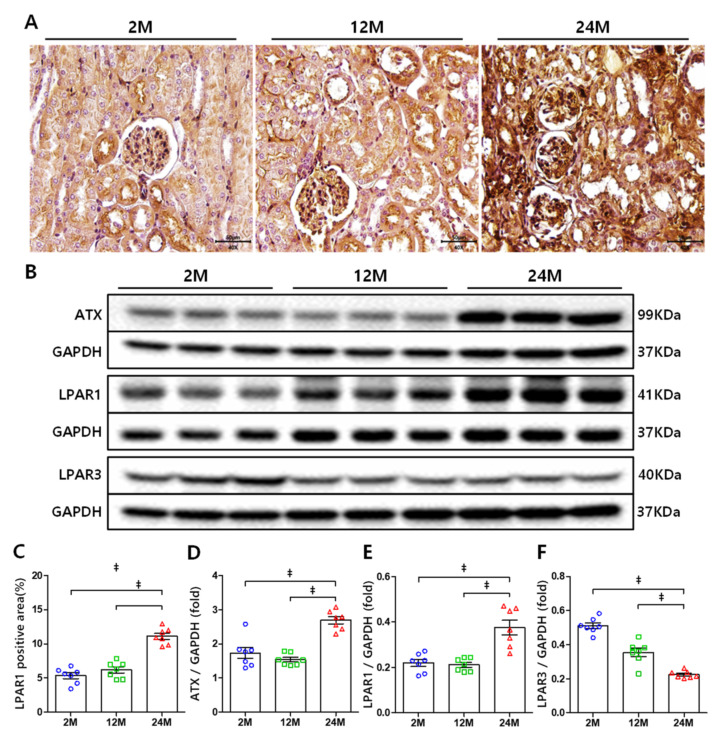
Renal expression of ATX, LPAR1, and LPAR3: (**A**) Representative image of IHC staining of LPAR1 in mice kidney (original magnification, × 400). (**B**) Representative Western blots of renal ATX, LPAR1, and LPAR3 protein levels. (**C**) The increased LPAR1 expression with age in IHC stain. (**D**) ATX expression increased in the 24 M group. (**E**) LPAR1 expression increased in the 24 M group. (**F**) LPAR3 expression decreased with age. ‡ *p* < 0.001.

To identify whether LPAR1 is expressed in proximal tubule, we performed immunohistochemistry. LPAR1 was expressed in both the proximal tubule and glomeruli, and its expression increased with age (Figure 2A,C). Western blotting showed that ATX and LPAR1 expression was highly increased in the 24 M group compared to that in the 2 M and 12 M groups (Figure 2B,D,E). However, the expression of LPAR3 was decreased in aged mice kidneys (Figure 2B,F).

### 3.3. PI3K, Akt, and NF-κB Expression Increased in the Kidneys of Aged Mice

**Figure 3:**
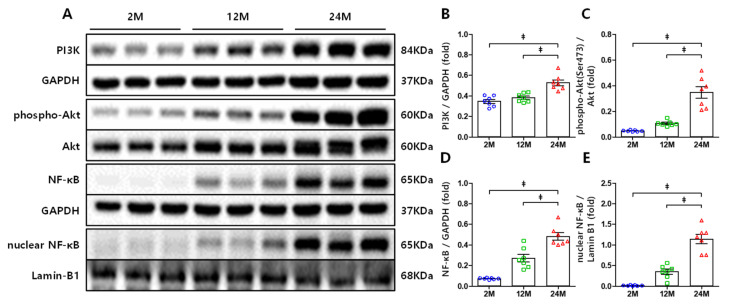
Renal expression of PI3K, phospho-Akt (Ser473), NF-κB, and nuclear-NF-κB: (**A**) representative Western blots of renal expression of PI3K, phospho-Akt (Ser473), NF-κB, and nuclear-NF-κB. (**B**) Compared to the 2 M and 12 M groups, the 24 M group exhibited a markedly increased PI3K expression. (**C**) Akt phosphorylation at Ser473 also considerably increased with age. (**D**) The NF-κB expression was significantly up-regulated in the 24 M group than the other groups (**E**) and the nuclear translocation of NF-κB increased accordingly. ‡ *p* < 0.001.

**Figure 4:**
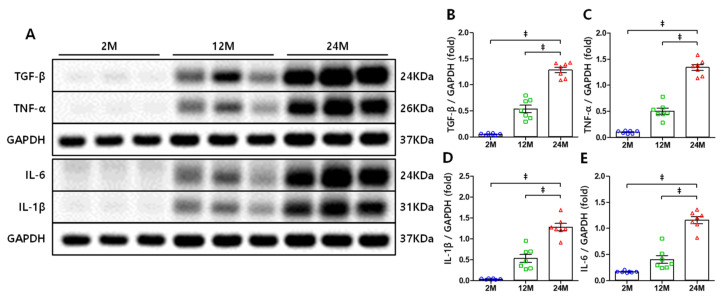
Renal expression of TGF-β, TNF-α, IL-1β, and IL-6: (**A**) Representative Western blots depicting TNF-α, IL-1β, IL-6, and TGF-β expression levels in mice kidney. (**B**) TGF-β expression greatly increased in the 24 M group, and similar increases were observed in (**C**) TNF-α, (**D**) IL-6, and (**E**) IL-1β expression. ‡ *p* < 0.001.

Phosphoinositide 3-kinases (PI3K)/Akt signaling is related to inflammation and fibrosis [22,35,36] and is capable of inducing NF-κB activation via the canonical pathway [37,38]. Western blot analysis was performed to assess the expression of these proteins in each group. Compared to the 2 M and 12 M groups, the 24 M group exhibited increased PI3K expression (Figure 3A,B) and, consequently, Akt phosphorylation at Ser473 also considerably increased (Figure 3A,C). NF-κB expression was significantly upregulated in the 24 M group compared to that in the other groups (Figure 3A,D), and the nuclear translocation of NF-κB increased accordingly (Figure 3A,E). Additionally, the expression of the profibrogenic cytokine TGF-β remarkably increased in the 24 M group compared to that in the 2 M and 12 M groups (Figure 4A,B).

### 3.4. NF-κB-Mediated Increase in the Expression Levels of TNF-α, IL-1β, and IL-6

NF-κB is recognized as a key mediator of aging due to its induction of various inflammatory cytokines such as tumor necrosis factor-α (TNF-α), interleukin-1β (IL-1β), and interleukin-6 (IL-6), and cytokines are essential for cell senescence [16,19,20]. We detected the expression of these cytokines using Western blot analysis. The expression of TNF-α was markedly increased in the 24 M group compared to that in the 2 M and 12 M groups (Figure 4A,C). Meanwhile, the expression of IL-1β (Figure 4A,D) and IL-6 (Figure 4A,E) exhibited similar increases.

### 3.5. DOXO and H_2_O_2_ Treatment Induced Cellular Senescence Resulting in Increased LPAR1 and NF-κB Levels

**Figure 5:**
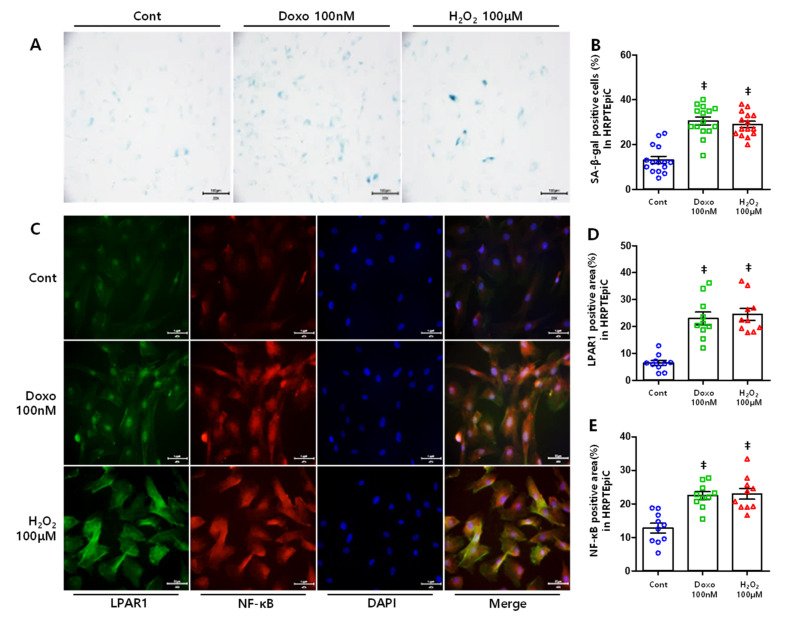
SA-β-gal stain and double immunofluorescence stain in senescence-induced cells: (**A**) representative image of SA-β-gal staining in senescence-induced HRPTEpiC; original magnification, ×200. (**B**) The HRPTEpiC treated with 100 nM DOXO and 100μM H_2_O_2_ showed increases in the number of SA-β-gal-positive cells. (**C**) Representative image of double immunofluorescence staining of LPAR1 and NF-κB in senescent HRPTEpiC; original magnification, ×200. (**D**) LPAR1- and (**E**) NF-κB-positive area increased in senescence-induced HRPTEpiC. ‡ *p* < 0.001.

**Figure 6:**
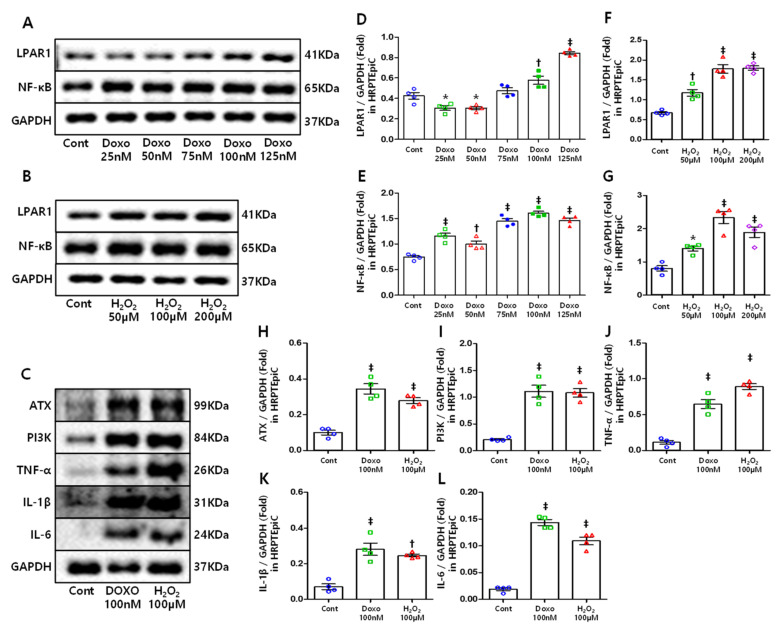
The ATX, LPAR1, PI3K, NF-κB, and inflammatory cytokines expression in senescent HRPTEpiC: (**A**–**C**) Representative Western blots demonstrating ATX, LPAR1, PI3K, NF-κB, and inflammatory cytokines expression in senescent cells. (**D**,**E**) LPAR1 and (**F**,**G**) NF-κB expression increased with DOXO and H_2_O_2_ treatment. (**H**–**L**) The ATX, PI3K, and inflammatory cytokines expression up-regulated in senescence-induced HRPTEpiC. * *p* < 0.05, † *p* < 0.01, ‡ *p* < 0.001.

In the present study, we simulated cell senescence conditions by triggering DNA damage and oxidative stress using doxorubicin and hydrogen peroxide, respectively [39,40]. HRPTEpiCs were treated with different doses of DOXO (25, 50, 75, 100, and 125nM) and H_2_O_2_ (50, 100, and 200 μM), and the expression of LPAR1 and NF-κB was determined by Western blotting. LPAR1 and NF-κB expression showed statistically significant increases with DOXO (Figure 6A,D,E) and H_2_O_2_ (Figure 6B,F,G) treatment. We then observed cell senescence using SA-β-gal stain [41]. HRPTEpiC treated with DOXO and H_2_O_2_ showed an increase in the number of SA-β-gal-positive cells (Figure 5A,B). In addition, senescence-induced cells were co-stained with LPAR1 and NF-κB, which resulted in increased LPAR1- and NF-κB-positive cells (Figure 5A,D,E).

### 3.6. ATX, PI3K, and Inflammatory Cytokines Increased in Senescent Cells

Normally, ATX induction in the tissue repair process is negatively regulated by LPA via LPAR1/PI3K signaling at the mRNA level. Subsequently, the induced ATX is rapidly eliminated in the liver. Therefore, once inciting injuries are resolved, the ATX returns to basal expression. However, if the injuries persist, the above regulation will be suppressed by excessive inflammatory cytokines, and ATX expression will continue to be high [21]. Western blotting was performed to determine whether ATX, PI3K, and inflammatory cytokines were increased in senescent cells. ATX expression was significantly increased in senescent HRPTEpiCs (Figure 6C,H). Meanwhile, PI3K (Figure 6C,I) and inflammatory cytokine (Figure 6C,J–L) expression also increased considerably and accordingly.

### 3.7. The LPAR1 Regulates NF-κB and Inflammatory Cytokines Expression Via PI3K, and NF-κB Is Also Essential in Maintaining the LPAR1 Level

**Figure 7:**
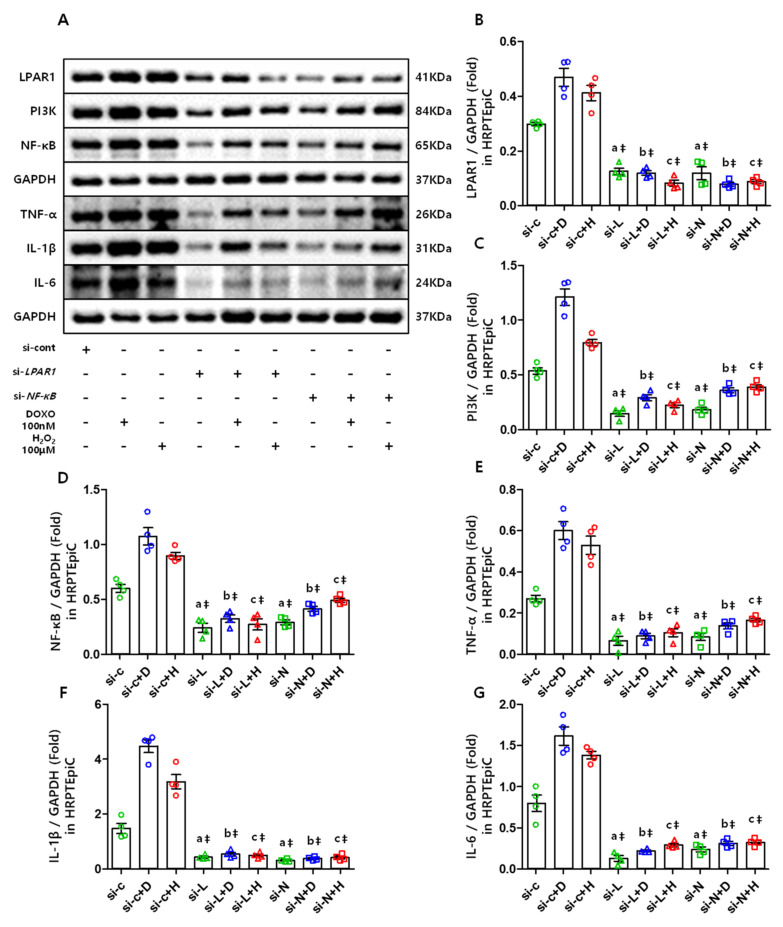
The expression of LPAR1, PI3K, NF-κB, and inflammatory cytokines in senescence-induced HRPTEpiC before and after si-*LPAR1* and si-*NF-κB* transfection: (**A**) representative Western blots of each protein involved. (**B**–**G**) LPAR1, PI3K, NF-κB, and inflammatory cytokine expression highly increased with senescence-induction of DOXO and H_2_O_2_ treatment, while si-*LPAR1* treatment reduced the increases. (a: si-c vs. si-L, si-N; b: si-c+D vs. si-L+D, si-N+H; c: si-c+H vs. si-L+H, si-N+H; si-c = si-control, si-L = si-*LPAR1*, si-N = si-*NF-κB*, D = doxorubicin, H = H_2_O_2_). ‡ *p* < 0.001.

To determine whether LPAR1 elevates NF-κB in senescence-induced cells, HRPTEpiCs were transfected with si-*LPAR1*, and then cultured in medium containing DOXO and H_2_O_2_. The results showed that knockdown of *LPAR1* (Figure 7A,B) markedly reduced NF-κB (Figure 7A,D) and inflammatory cytokines (Figure 7A,E–G) along with a decrease in PI3K (Figure 7A,C). Moreover, to confirm the association between NF-κB and LPAR1, we transfected si-*NF-κB* into HRPTEpiC and induced senescence in the same manner. The results showed that not only did the NF-κB (Figure 7A,D) and inflammatory cytokine (Figure 7A,E–G) expression decrease, but LPAR1 (Figure 7A,B) and PI3K (Figure 7A,C) decreased as well. This suggests that LPAR1 and NF-κB are interdependent in maintaining high expression levels via PI3K during cellular senescence.

## 4. Discussion

Progressive loss of physiological integrity is a characteristic of aging [2]. Lopez-Otin et al. [1] reported that aging is a consequence of a gradual accumulation of cell damage that may result in the loss of cellular fitness. In the present study, we compared male 2-, 12-, and 24-month-old C57BL/6 mice. The aged kidney showed decreased renal function, increased fractional mesangial area, and tubulointerstitial fibrosis. We identified elevated inflammatory cytokines that were mediated by increased ATX, LPAR1, PI3K, and NF-κB expression in aged kidneys, whereas LPAR3 decreased. In addition, knockdown of *LPAR1* in senescence-induced cells decreased NF-κB and subsequent inflammatory cytokine induction, and knockdown of *NF-κB* resulted in reduced LPAR1 expression.

Cellular senescence occurs in response to various injuries and plays a pivotal role in tissue repair [25]. Qi and Yang [36] reported that maladaptive repair may induce senescence in TECs, which are responsible for renal aging [24,25,26]. The ATX/LPA axis is associated with wound healing, tissue remodeling, and chronic inflammation [42]. LPA is a small ubiquitous lipid that is mainly converted by the phospholipase D activity of ATX in the extracellular space. ATX is widely expressed, with high mRNA levels detected in kidneys, and is also detected in most biological fluids [43,44]. Of note, it has been reported that LPA and ATX have a negative feedback mechanism in which its product, LPA, may inhibit ATX activity; however, the feedback loop is not successful in the case of chronic inflammation [42,45]. According to these studies, inflammatory cytokines (e.g., TNF-α, IL-1β, and IL-6) from damaged tissue stimulate ATX expression and subsequent LPA production for wound healing. In this scenario, the wound recovers and inflammation subsides, and ATX is eventually downregulated by LPA. However, when the insult persists, the negative feedback mechanism of LPA to ATX is disturbed by the continuous generation of inflammatory cytokines, and LPA induction increases continuously, which in turn causes a further release of inflammatory cytokines. In this study, we identified markedly increased ATX levels in aged mice, proving that ATX/LPA axis-mediated inflammation is related to renal aging.

The various biological effects of LPA are exerted by binding to its receptors. In the present study, we found that LPAR1 is mainly expressed in the glomeruli and proximal tubules, which is consistent with the findings of other researchers [27]. Meanwhile, Western blot analysis showed highly increased LPAR1 and cytokines in the aged kidney, which decreased with si-*LPAR1* treatment in senescence-induced cells. This suggests that LPAR1 mediates the induction of inflammatory cytokines in TECs. In contrast to the increase in LPAR1, LPAR3 was decreased in aged kidneys. Chen et al. [46] reported that LPAR3 prevents oxidative stress and cellular senescence in Hutchinson–Gilford progeria syndrome. Considering the pivotal role of TECs in renal aging [24,25,26], blocking LPAR1 may prevent cellular senescence, or even attenuate renal aging. While LPAR3 seems to have a potential role in anti-aging, subsequent in vivo experiments are needed to further confirm its role.

NF-κB is recognized as a key mediator of aging [16,19,20], and its activity is necessary for the development of aging features [47]. It has been reported that senescence-inducing ionizing radiation increases the secretion of inflammatory cytokines from cells. In turn, secreted cytokines activate NF-κB, which may subsequently stimulate further inflammatory cytokine induction. If the immune system fails to eliminate senescent cells, the constantly reinforcing positive feedback mechanism will eventually result in a senescence load [16,48]. Many researchers have reported that PI3K signaling induces inflammatory cytokine production by activating NF-κB through downstream Akt [37,38], and continuously activated PI3k signaling may result in cellular senescence [49]. Our results showed increased PI3K and NF-κB expression in the aged kidney, and TNF-α, IL-1β, and IL-6 expression markedly increased. In contrast, si-LPAR1-treated cells showed a reduction in PI3K, NF-κB, and inflammatory cytokines, which confirmed that NF-κB expression was under the control of LPAR1 signaling. Moreover, si-*NF-κB*-treated cells showed decreased NF-κB and inflammatory cytokine expression, as well as LPAR1. This finding suggests that not only can LPAR1 induce NF-κB expression, but NF-κB is also necessary for maintaining a continuously increased LPAR1. Interestingly, Zhao et al. [44] reported a negative LPAR1 regulation mechanism in their review that LPA/LPAR1 binding may trigger LPAR1 lysosomal degradation, however if it fails, it may promote an LPA/LPAR1 mediated inflammatory response. The role of the LPAR1 regulation mechanism in the aging process is unclear, yet it seems to be broken down during aging, and one thing that we can be sure of is the existence of a mutual reinforcing mechanism between aberrantly elevated LPAR1 and NF-κB.

**Figure 8:**
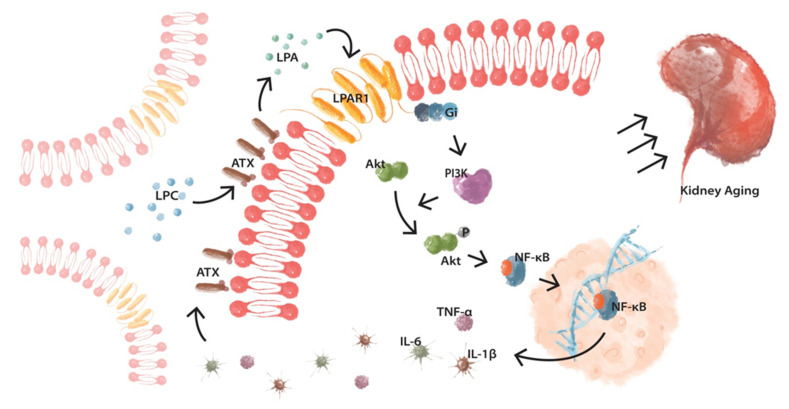
Cartoon summarizing aberrantly activated LPAR1 signaling mediates excessive inflammatory response via continually reinforcing loop in renal aging.

These data suggest that LPAR1 signaling initially mediates the repair process upon cellular damage. Meanwhile, a series of negative feedback mechanisms ensures that the inflammatory response will not be over-activated. However, these mechanisms seem to fail upon sustained insults, and eventually, aberrantly activated LPAR1 signaling leads to the propagation of inflammation, contributing to aging (Figure 8). Many cellular-damaging factors described previously cannot be completely avoided to date. Therefore, blocking aberrantly activated LPAR1 may be a novel therapeutic strategy to attenuate renal aging.

## 5. Conclusions

Our study revealed that aberrantly activated LPAR1 signaling mediates inflammation in aged kidneys. In addition, we identified that signaling is involved in the continuous reinforcement of inflammatory responses. Therefore, blocking aberrantly activated LPAR1 may reduce excessive inflammation, which provides a new possible therapeutic strategy to attenuate renal aging.

## Data Availability

The data presented in this study are available in this article.
